# The Influence of Abstinence Interval on Semen Quality and Its Effect on an Assisted Reproductive Technology (ART) Pregnancy: A Case Report

**DOI:** 10.7759/cureus.54226

**Published:** 2024-02-15

**Authors:** Rokaiya Shaikh, Akash More, Shilpa Dutta, Namrata Choudhary, Shivani Khemani

**Affiliations:** 1 Clinical Embryology, Datta Meghe Institute of Higher Education and Research, Wardha, IND

**Keywords:** scd, pregnancy, art, asthenoteratozoospermia, icsi, dna fragmentation

## Abstract

This case report focuses on a couple facing primary infertility, where the male partner exhibited asthenoteratozoospermia and high DNA fragmentation. The treatment approach involved three cycles of intracytoplasmic sperm injection (ICSI), an assisted reproductive technology (ART), to indicate and address the specific challenges posed by male factor infertility. The initial two attempts failed as DNA fragmentation was high, which was observed on days 4 and 3 of abstinence, respectively. In the third cycle, DNA fragmentation was low on day 2 of the abstinence period, resulting in the successful formation and cryopreservation of embryos. Subsequently, three months later, frozen embryo transfer (ET) was done. This was followed by a positive β-human chorionic gonadotropin (hCG) test after 14 days that confirmed biochemical pregnancy, and successful conception was determined by ultrasound detection of the visible sac with fetal pole.

This report underscores the critical importance of treatment plans for individual patients, especially considering the impact of abstinence periods on sperm DNA fragmentation. The findings promote a personalized approach to assisted reproductive techniques, enhancing the success rate. It is recommended that further comprehensive studies be conducted to validate and anticipate these observations.

## Introduction

The reproductive structures, pivotal for human procreation, are integral components of the male reproductive system [1]. Male infertility denotes the inability of a man to contribute to conception due to complications within his reproductive anatomy or physiology. Infertility is operationally defined as the failure of a couple to achieve conception after one year of regular, unprotected intercourse. Comprehensive evaluation and management of infertility encompasses both male and female partners and takes into consideration factors such as age, medication, surgery, environmental exposures, genetic predispositions, systemic diseases, and multifactorial etiologies contributing to male infertility, including environmental toxins such as insecticides, fungicides, pesticides, and substance abuse [2].

Fragmentation of DNA entails the disruption or cleavage of DNA molecules into distinct fragments. Sustaining sperm DNA integrity is fundamental for successful fertilization and the production of viable offspring. This DNA fragmentation may occur as a result of various factors, including oxidative stress, impaired maturation processes, and aberrant programmed cell death pathways, all of which are disproportionately encountered in assisted reproductive techniques. Clinical and environmental variables significantly influence sperm DNA fragmentation dynamics. Extrinsic factors such as smoking, heat exposure, and environmental pollutants contribute to DNA fragmentation, while intrinsic factors including cellular maturation processes, germ cell dysfunction, defective apoptosis, and oxidative stress contribute to its occurrence. Different types of DNA damage, such as modifications, cross-links, pyrimidine dimers, single-strand breaks (SSBs), and double-strand breaks (DSBs), are observed. The DNA fragmentation tends to increase with advancing age, correlating with heightened exposure to oxidative stress, compromised sperm quality, and dysregulated apoptosis [3].

The efficacy of assisted reproductive technology (ART) in treating infertility is notably influenced by sperm quality. Asthenozoospermia, characterized by a reduced total motile sperm count (less than 40%) and progressive motility (less than 32%), poses challenges to natural conception [4]. Intracytoplasmic sperm injection (ICSI) is a technique that circumvents compromised sperm motility by directly injecting a single sperm into the oocyte cytoplasm. Asthenozoospermic specimens constitute a significant proportion of the substrates utilized in contemporary ART procedures [4].

This case report focuses on a patient diagnosed with asthenoteratozoospermia, characterized by impaired sperm motility and morphology coupled with elevated levels of DNA fragmentation. The investigation aims to determine the optimal abstinence timing for semen collection in such cases.

## Case presentation

Medical history

The couple presented with a history of infertility spanning seven years, with the female partner aged 35 and the male partner aged 37. The male, employed as a farmer, and the female, a homemaker, exhibited distinct medical backgrounds. The female had undergone a cholecystectomy in 2018 and was concurrently managing hypothyroidism with medication. 

The male had previously experienced kidney stone episodes, was managed pharmacologically, and had a history of tuberculosis. Additionally, both individuals had a familial predisposition to hypertension. Notably, there were no reported familial tendencies toward alcohol consumption, smoking, or tobacco usage.

Prior clinical encounters included two unsuccessful attempts at intrauterine insemination (IUI) and two failed cycles of ICSI in 2019, following a four- to five-day abstinence period. The patient was diagnosed with primary infertility and had previously sought consultation at a facility located in Sawangi, Wardha, Maharashtra, India.

Clinical findings

Upon their visit to the fertility clinic, a thorough assessment was conducted to identify the causes of infertility. In this scenario, the focus was on the male partner's semen analysis. The results indicated a condition known as asthenoteratozoospermia, characterized by abnormal sperm motility and morphology.

After analyzing the semen sample provided by the male partner, certain deviations from the normal parameters were observed. The volume of the semen was within the standard range of 2 milliliters. However, the sperm count was measured at 17 million sperm per milliliter.

The motility of the sperm, which referred to their ability to move effectively, was 28%. Additionally, there was a high percentage of defective sperm, with 97% showing abnormalities in their structure after the four-day abstinence period. This condition is referred to as asthenoteratozoospermia.

Treatment

The couple sought treatment at the infertility center due to difficulties conceiving. Following four days of abstinence, semen analysis of the male partner revealed a normal sperm count of 17 million/ml, although morphological assessments were not satisfactory for the procedure. Subsequently, ovum pickup was conducted to retrieve oocytes, followed by ICSI on the same day. Of the five oocytes retrieved, comprising two metaphase I (MI), two MII, and one germinal vesicle (GV) grade oocyte, a fertilization check after 15 to 18 hours yielded negative results, as no pronuclei were detected, thus resulting in the failure of ICSI. The sperm DNA fragmentation (SDF) was done in this cycle, and it was around 31%.

In the subsequent cycle of ovum pickup, six oocytes were retrieved, consisting of two MI and three MII, alongside one GV-grade oocyte. A fresh semen sample was collected on the third day of abstinence for SDF analysis, which revealed over 25% DNA fragmentation, with hollow DNA fragmentation ranging from 35% to 39%. Despite the ICSI procedure performed on the same day, it failed due to genetic abnormalities.

**Figure 1:**
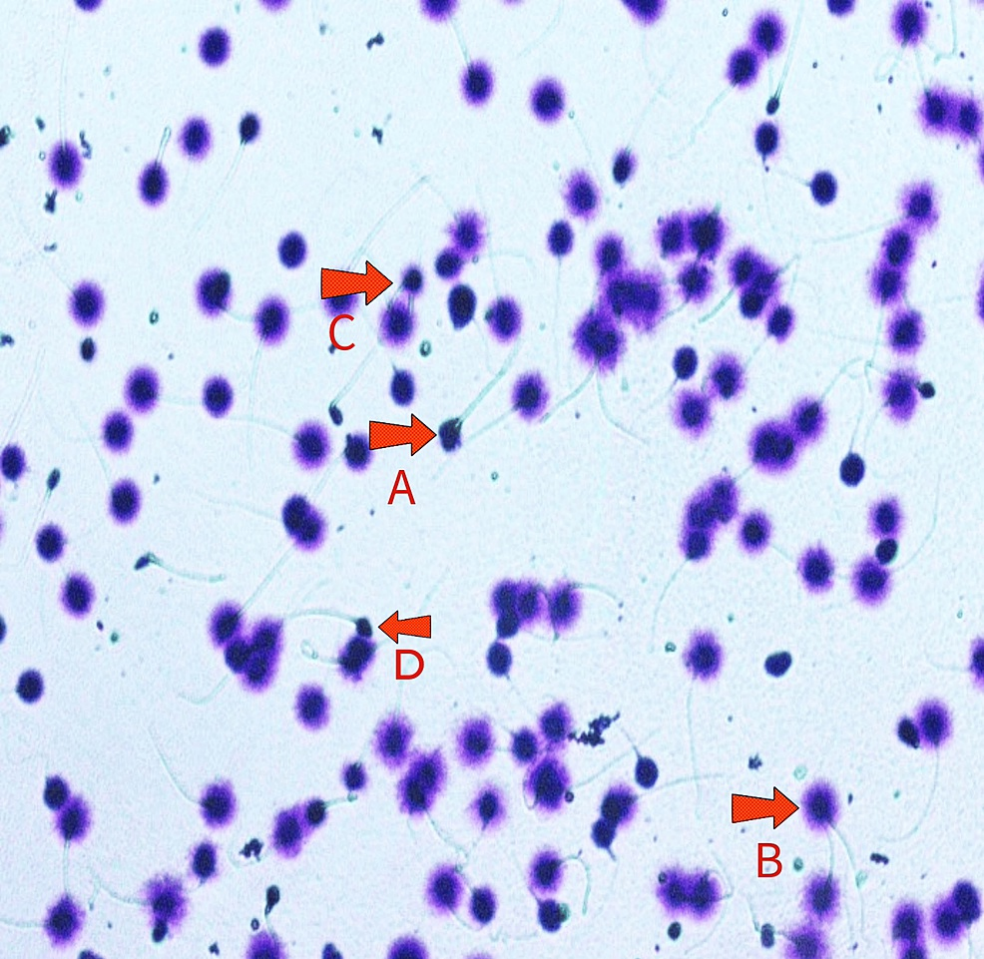
Sperm DNA chromatin assay of the patient A: Medium halo on sperm head, B: Big halo on sperm head, C: Small halo on sperm head, D: Fragmented DNA

Three oocytes were retrieved during the third cycle of ovum pickup, including two MII and one MI. A semen sample was collected after a two-day abstinence interval and showed reduced sperm DNA fragmentation (17%) (Figure 1), attributed to the variance in abstinence duration. Following ICSI, two embryos were successfully formed and subsequently cryopreserved. Embryo transfer (ET) was scheduled for three months later. On the day of ET, both embryos were thawed and transferred after a two-hour interval.

Follow up

Following ET, the female patient received recommendations to adhere to a nutritious diet, refrain from heavy lifting, and prioritize rest. Fourteen days post-ET, a β-human chorionic gonadotropin (hCG) test was conducted, confirming pregnancy with a test value of 1225 mIU/mL. She was advised to supplement her diet with multivitamins and abstain from strenuous activities to support the ongoing pregnancy.

## Discussion

Pons et al. illustrated that adherence to a particular day of abstinence elicits a reduction in sperm DNA fragmentation in 90% of the chosen subjects. A cohort comprising 416 patients who refrained from sexual activity for a period ranging from three to seven days underwent examination. Among them, 35 patients with elevated DNA fragmentation levels underwent a one-day abstinence regimen, which subsequently led to diminished fragmentation levels after a span of three days [5]. Magnus et al. investigated the impact of varying abstinence periods on sperm quality in the same individuals, suggesting a potential benefit in advising longer abstinence periods, up to 10 days, before semen collection [6].

Vanessa et al. conducted a study on the influence of abstinence periods on human sperm quality, enrolling 2458 men undergoing infertility evaluation. Their findings underscored the detrimental effect of prolonged abstinence on DNA damage, likely attributable to reactive oxygen species (ROS). This study served as the foundation for our investigation [7]. Li et al. investigated the effect of male sexual abstinence periods on the clinical outcomes of fresh ET. Meta-analysis results indicated that shorter abstinence periods were correlated with higher implantation and pregnancy rates in patients undergoing ART treatment [8]. In our study, a shorter abstinence period resulted in a lower DNA fragmentation index (DFI) and successful conception.

Male infertility diagnosis traditionally relies on microscopic examination of sperm concentration, motility, and morphology in ejaculate samples, which is crucial for assessing sperm quality [9]. Sperm DNA fragmentation is a major contributor to male infertility, attributed to apoptosis, ROS, and protamination failures. Routine semen analysis cannot detect sperm chromatin damage, underscoring the importance of sperm DNA fragmentation testing in diagnosing reproductive issues due to its impact on sperm functionality [10]. The limitation of our case report lies in its single-patient focus, suggesting the need for further investigations to validate these findings.

## Conclusions

In summary, our patients, a couple experiencing primary infertility, underwent three cycles of ICSI at our fertility center. The initial two cycles proved unsuccessful, with embryo formation impeded by elevated DNA fragmentation levels observed during days 4 and 3 of abstinence. However, the third ICSI cycle yielded success, resulting in embryo formation, which was subsequently cryopreserved. Reduced DNA fragmentation during this cycle was attributed to a shorter abstinence period of two days. The couple was advised to undergo frozen ET, which was scheduled three months post-cryopreservation. Following ET, a positive β-hCG test at 14 days confirmed successful conception.

This report underscores the crucial role of personalized treatment plans tailored to individual patients, particularly considering the impact of abstinence periods on sperm DNA fragmentation. These findings advocate for a shorter period of abstinence for ART, thereby enhancing success rates. Further comprehensive studies are recommended to validate and anticipate these observations.
